# Delivery of thymoquinone to cancer cells with as1411-conjugated nanodroplets

**DOI:** 10.1371/journal.pone.0233466

**Published:** 2020-05-21

**Authors:** Emily M. Murphy, Connor S. Centner, Paula J. Bates, Mohammad T. Malik, Jonathan A. Kopechek

**Affiliations:** 1 Department of Bioengineering, University of Louisville, Louisville, Kentucky, United States of America; 2 Department of Medicine, University of Louisville, Louisville, Kentucky, United States of America; 3 Molecular Targets Program of the James Graham Brown Cancer Center, University of Louisville, Louisville, Kentucky, United States of America; 4 Department of Microbiology and Immunology, University of Louisville, Louisville, Kentucky, United States of America; Universidade Nova de Lisboa, PORTUGAL

## Abstract

Systemic delivery of conventional chemotherapies can cause negative systemic toxicity, including reduced immunity and damage to organs such as the heart and kidneys—limiting the maximum dose that can be administered. Targeted therapies appear to address this problem by having a specific target while mitigating off-target effects. Biocompatible perfluorocarbon-based nanodroplet emulsions encapsulated by a phospholipid shell are in development for delivery of molecular compounds and hold promise as vehicles for targeted delivery of chemotherapeutics to tumors. When ultrasound is applied, perfluorocarbon will undergo a phase change—ultimately inducing transient perforation of the cell membrane when in close proximity, which is more commonly known as “sonoporation.” Sonoporation allows enhanced intracellular delivery of molecular compounds and will reseal to encapsulate the molecular compound intracellularly. In this study, we investigated delivery of thymoquinone (TQ), a natural hydrophobic phytochemical compound with bioactivity in cancer cells. In addition, we conjugated a G-quadruplex aptamer, ‘AS1411’, to TQ-loaded nanodroplets and explored their effects on multiple human cancer cell lines. AS1411 binds nucleolin, which is over-expressed on the surface of cancer cells, and in addition to its tumor-targeting properties AS1411 has also been shown to induce anti-cancer effects. Thymoquinone was loaded onto AS1411-conjugated nanodroplet emulsion to assess activity against cancer cells. Confocal microscopy indicated uptake of AS1411-conjugated nanodroplets by cancer cells. Furthermore, AS1411-conjugated nanoemulsions loaded with TQ significantly enhanced cytotoxicity in cancer cells compared to free compound. These results demonstrate that AS1411 can be conjugated onto nanodroplet emulsions for targeted delivery to human cancer cells. This novel formulation offers significant potential for targeted delivery of hydrophobic chemotherapeutics to tumors for cancer treatment.

## Introduction

Standard clinical treatments for cancer patients include surgery, radiation, and chemotherapy. Administration of chemotherapeutic drugs has been used for cancer treatment since the 1940s but targeted anti-cancer therapies have only recently been developed [1, 2]. Currently, conventional chemotherapy drugs are typically delivered systemically and induce adverse effects in other organs, including reduced immune activity and damage to organs such as the heart and kidneys [3]. Therefore, the therapeutic window that can be administered is limited. To address this problem, targeted delivery strategies are in development to increase the efficacy of chemotherapy while reducing negative systemic toxicity.

**Fig 1:**
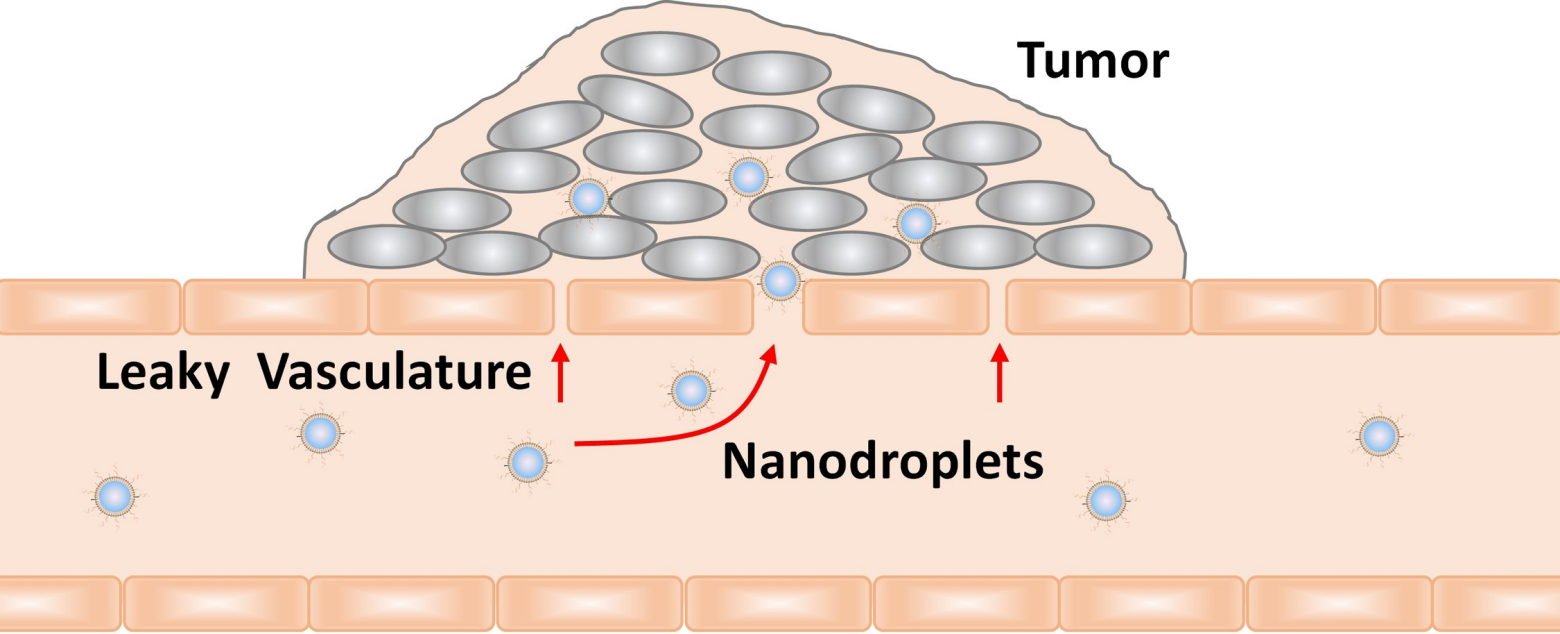
Illustration of nanodroplet accumulation in tumors via the enhanced permeability and retention effect.

To overcome limitations with systemic intravenous delivery of chemotherapeutic drugs, perfluorocarbon-based nanodroplet emulsions have been developed which consist of liquid nanodroplets with average diameters of 100–400 nm. The nanodroplets are composed of a biocompatible phospholipid shell encapsulating an inert, non-toxic perfluorocarbon such as perfluoropentane (PFP). Hydrophobic drugs can be incorporated within the lipid shell for transport through circulation [4]. Nanoemulsions accumulate in tumors via the enhanced permeability and retention (EPR) effect, which occurs due to the inherently leaky vascular structure of tumors (Fig 1) [5–8]. The nanodroplets can enter the cells via receptor-mediated endosomal pathways where their components are metabolized and the drugs are released [9]. To facilitate receptor-mediated uptake, targeting moieties, such as aptamers, can be conjugated to the nanodroplet phospholipid membrane [10]. Furthermore, the liquid perfluorocarbon in nanoemulsions can be vaporized into gas bubbles using ultrasound or light, which enhances molecular delivery to targeted cells. Following vaporization, the inert perfluorocarbon is cleared through exhalation [11–15].

In this study, we report for the first time a novel nanoemulsion formulation consisting of thymoquinone-loaded perfluorocarbon nanodroplets conjugated with a tumor-targeting aptamer, AS1411. This aptamer is a 26-mer guanine-rich DNA oligonucleotide that has been found to form G-quadruplex structures and bind to nucleolin, which is preferentially expressed on the surface of cancer cells [16]. AS1411 has been shown to induce cancer cell cytotoxicity by causing methuosis, a novel type of non-apoptotic cell death, via nucleolin-stimulated Rac1 activation [17]. Therefore, AS1411 potentially serves as both a tumor-targeting agent and an anti-cancer therapeutic agent. AS1411 has been evaluated in clinical trials but enhanced stability in circulation is needed to improve efficacy [18]. Recently, AS1411 has been conjugated to multiple types of nanoparticles for enhanced tumor uptake in preclinical studies [16, 19–21]. However, these nanoparticles lack the ability to load chemotherapeutic compounds inside their vesicle. Therefore, the objective of this study was to evaluate AS1411-conjugated nanodroplet emulsions for targeted delivery of molecular compounds to cancer cells. In this study we loaded thymoquinone (TQ), a natural hydrophobic phytochemical compound with bioactivity in cancer cells, in AS1411-conjugated nanodroplets and investigated their effect on human breast cancer cells. Thymoquinone is derived from the *Nigella sativa* plant and has been reported to have beneficial effects including diuretic, hypotensive, anti-histaminic, anti-inflammatory, analgesic, anti-epileptic, and potent anti-tumor effects [22, 23]. However, the strong hydrophobicity of thymoquinone has limited its clinical translation. By loading thymoquinone into AS1411-conjugated nanodroplets, targeted tumor delivery can potentially be achieved. This study was aimed at establishing a proof-of-principle on the path toward potential future clinical translation of this therapeutic formulation.

## Materials and methods

### Synthesis of AS1411-conjugated drug-loaded nanodroplets

**Fig 2:**
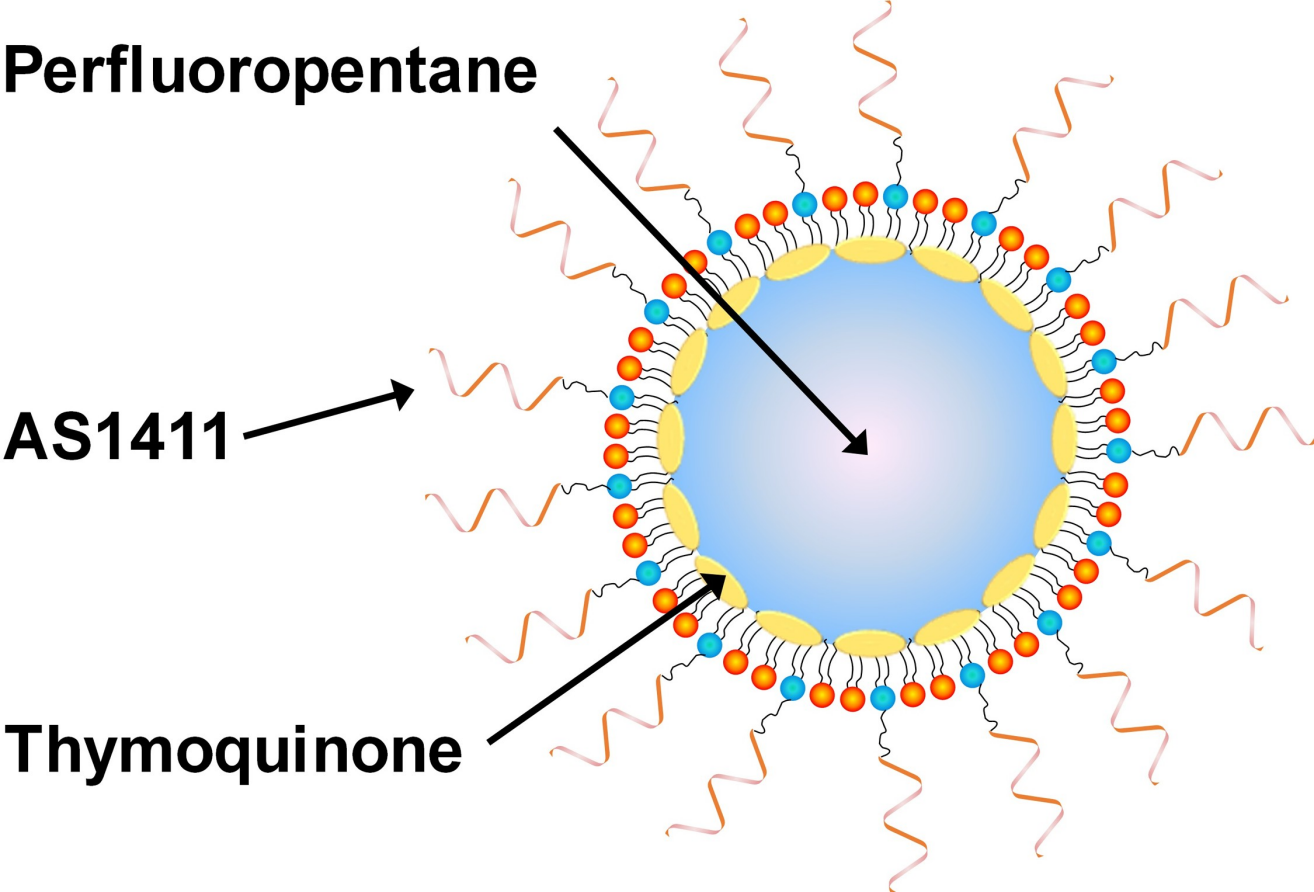
Schematic of AS1411-conjugated nanodroplets loaded with thymoquinone (not to scale).

AS1411-conjugated nanodroplets loaded with thymoquinone were synthesized. The nanodroplets were composed of a perfluorocarbon core surrounded by a lipid shell. Thymoquinone, which is hydrophobic molecule, incorporates within the lipid shell and thiolated AS1411 was conjugated to maleimide-lipids (Fig 2). Phospholipids were obtained from Avanti Polar Lipids (Alabaster, AL, USA). Nanodroplets were composed of 1,2-dipalmitoyl-sn-glycero-3-phosphocholine (DPPC), 1,2-distearoyl-sn-glycero-3-phosphoethanolamine-N-[amino(polyethylene glycol)-2000] (DSPE-PEG2000), and 1,2-distearoyl-sn-glycero-3-phosphoethanolamine-N-[maleimide(polyethylene glycol)-2000] (DSPE-PEG2000-maleimide) in a 96:2:2 molar ratio. For fluorescent studies 1,2-Distearoyl-sn-Glycero-3-Phosphoethanolamine (DSPE-PEG2000-FITC) was added instead of DSPE-PEG2000. Perfluorocarbon nanoemulsions were synthesized following a procedure used in previous studies with modifications to enable AS1411 conjugation and thymoquinone loading [24]. Lipids were dissolved in chloroform and the solvent was evaporated under argon. The dry lipid film was rehydrated in phosphate buffered saline (PBS) to a concentration of 2.3 mg/mL and stored at 4°C until use. Thymoquinone (Sigma-Aldrich, St. Louis, MO, USA) was added to the lipid solution at a concentration of 8 mg/mL and sonicated at 60% amplitude for 30 seconds with a sonicator (Qsonica, Newtown, CT, USA) to disperse the drug and lipid. Aptamers were obtained from IDT Technologies (Coralville, IA, USA). Sequences consisted of AS1411 (5′-GGTGGTGGTGGTTGTGGTGGTGGTGG-3′) and CRO, the negative control (5′-CCTCCTCCTCCTTCTCCTCCTCCTCCT-3′) with thiol groups attached to the 3′ end. Aptamers (50 μM) were deprotected with 10 mM of (tris(2-carboxyethyl)phosphine) (TCEP) for 1 hour and immediately added to lipid solutions for overnight incubation at 4°C to allow conjugation of aptamers to lipid via thiol-maleimide reaction.

To produce nanodroplets, perfluoropentane (Fluoromed, Round Rock, TX, USA) was added to lipid solutions at 40% v/v and sonicated at 60% amplitude in an ice bath for 5 minutes in pulsed mode (20 s on-40 s off, 100 s total sonication duration). Following sonication, the emulsion was centrifuged at 2000 *g* for 3 min and the supernatant was aspirated to remove lipid/drug/aptamer not bound to droplets. The pellet of droplets was resuspended and diluted 5-fold in PBS, followed by extrusion 10 times through a 0.2-μm membrane (Mini-extruder, Avanti Polar Lipids). Absorbance measurements (NanoDrop One, Thermo Scientific, Waltham, MA, USA) indicated a lipid concentration of 200 μg/ml and an AS1411 concentration of 0.1 μM on the nanoemulsion. Based on the lipid concentration and the size of the nanodroplets, it was estimated that 0.04 AS1411 particles per nm^2^ are bound to each nanodroplet. The size distribution of the nanoemulsion was measured using a Particle Size Analyzer (Brookhaven Instruments, Holtsville, NY, USA). Thymoquinone loading was quantified with a NanoDrop One using the absorbance at 260 nm after subtracting the contribution from lipids and nanodroplets alone on the absorbance.

### Microscopy imaging of cellular uptake

Fluorescent microscopy uptake studies were conducted using FITC-labeled, AS1411-conjugated nanodroplets. Images were acquired using an EVOS FL digital fluorescence microscope (Advanced Microscopy Group, Mill Creek, WA, USA). Human MDA-MB-231 breast cancer cells were plated for 48 hr at a density of 4,000 cells/cm^2^ in glass cell culture dishes (FluoroDish, World Precision Instruments, Sarasota, FL USA). AS1411-conjugated fluorescent nanodroplet emulsions were added to cells at various doses (4%, 2%, 1%, 0.4%, 0.2% and 0% v/v PFP) and incubated for various amounts of time (0, 1, 4, 24, 48, and 72 hr) at 0.4% v/v PFP. Slides were washed with HBSS, fixed with 3.5% paraformaldehyde, stained with 0.05% Hoechst 33342 for 5 minutes at room temperature to detect nuclei, washed twice, and mounted (ClearMount, Invitrogen, Frederick, MD, USA) for at least 3 hours prior to imaging. All images were acquired with identical microscope settings (60% brightness for FITC and 10% brightness for Hoechst). Fluorescence intensity of FITC in cells was quantified using ImageJ.

### Confocal imaging of cellular uptake

Confocal microscopy uptake studies were conducted using Cy5-AS1411-conjugated nanoemulsions with FITC-labeled lipids. Images were acquired using a Nikon confocal microscope. Human triple negative breast cancer cells (MDA-MB-231) were plated for 48 hours at a density of 6,000 cells/dish in glass cell culture dishes (FluoroDish, World Precision Instruments, Sarasota, FL USA). The dishes were then treated with nanoemulsions (with or without AS1411) and incubated for 4 hr and 24 hr. Dishes were washed with HBSS, fixed with 3.5% paraformaldehyde for 15 minutes, stained with 0.05% Hoechst 33342 for 5 minutes at room temperature to detect nuclei, washed twice, and mounted (ClearMount, Invitrogen, Frederick, MD, USA) for at least 3 hours prior to imaging. All images were acquired with identical acquisition settings.

### Flow cytometry analysis

Flow cytometry studies were performed using FITC-labeled nanoemulsions (without Cy5) that were synthesized as above. MDA-MB-231 cells were plated in 35mm dishes at a density of 75,000 cells/well for 24 hours. Cells were treated with fluorescent nanoemulsions (with or without AS1411) for various amounts of time (1 h, 4 h, and 24 h). Samples were then washed with PBS, trypsinized, washed by centrifugation, and analyzed with a flow cytometer (MACSQuant, Miltenyi Biotec). Data was analyzed using flow cytometry software (FlowJo).

### In vitro cytotoxicity studies

Cytotoxicity of AS1411-conjugated drug-loaded nanoemulsions was tested in human breast cancer cells (MDA-MB-231 and HCC1395) using MTT assays. Cells were seeded in a 96-well plate at a concentration of 1000 cells/well and cultured for 48 h prior to treatment. Control groups consisted of no treatment, untargeted drug-loaded nanoemulsions, and free drug. Nanoemulsions were added to cell cultures at various concentrations and incubated for 48 h. MTT results were normalized to the no treatment control samples.

### Statistical analysis

Statistical comparisons between experimental and control groups were determined using a Student's t-test, with statistical significance defined as p < 0.05 (two-tailed). Comparisons between more than two groups were determined using ANOVA. Bars represent mean ± standard error.

## Results

### Characterization of nanoemulsions

**Fig 3:**
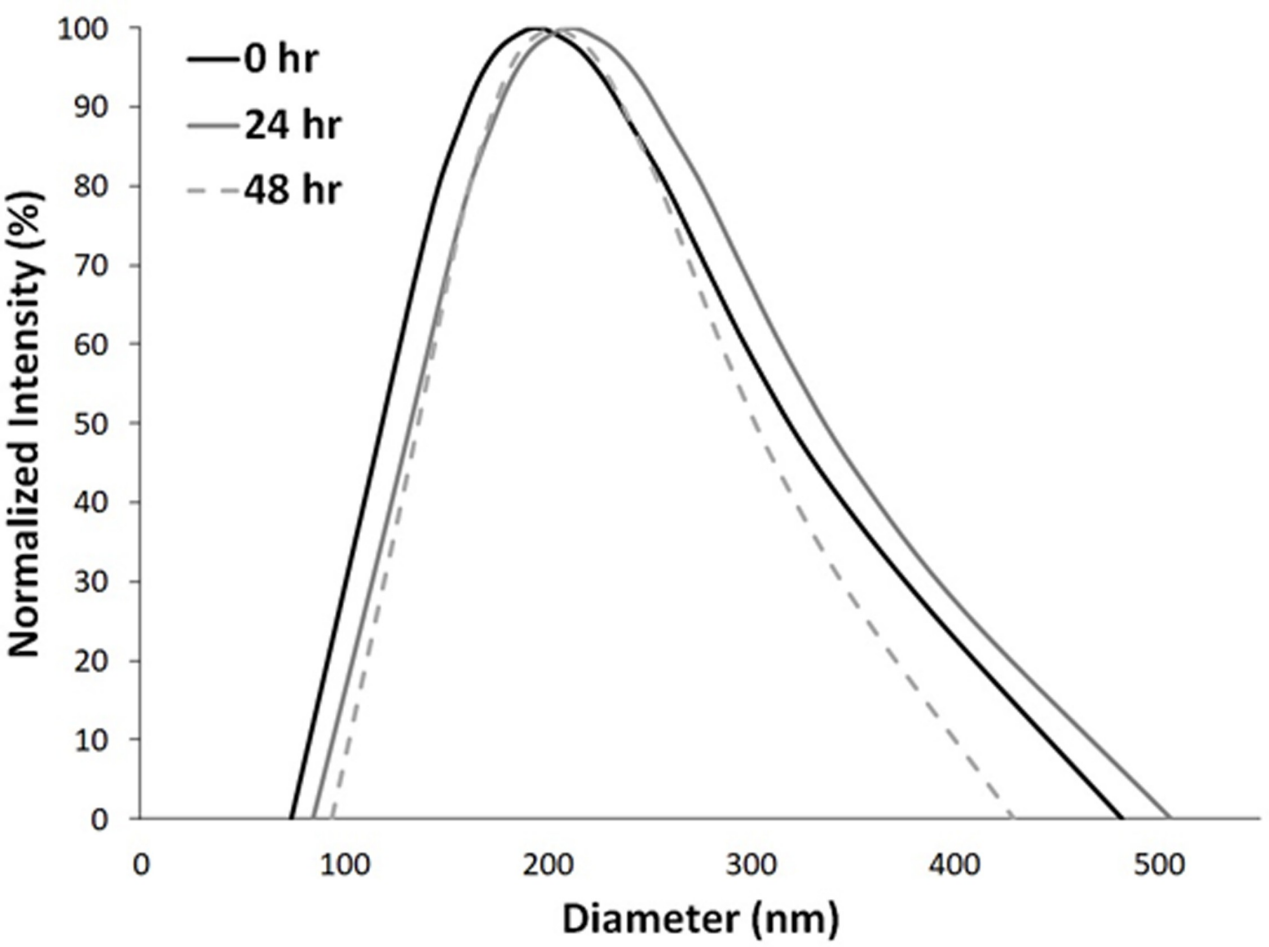
Dynamic light scattering measurements indicating minimal change in size distribution of AS1411-conjugated nanodroplets up to 48 hr.

The size distribution of nanodroplet emulsions was determined using dynamic light scattering. Although small changes in the average size were measured, no statistical difference was detected at each measured time point, indicating that the nanodroplets were stable for at least 48 h when stored at 4°C (Fig 3). In addition, the loading efficiency of thymoquinone in nanodroplets was determined using absorbance spectrometry. The thymoquinone concentration in the nanodroplet emulsion after extrusion and 5-fold dilution in PBS was calculated to be ~1 mM following the procedure described in the Methods, indicating a loading efficiency of ~10%. The total amount of thymoquinone in the 1-mL volume of nanodroplet emulsion was 1 μmol.

### Microscopy imaging of cellular uptake

**Fig 4:**
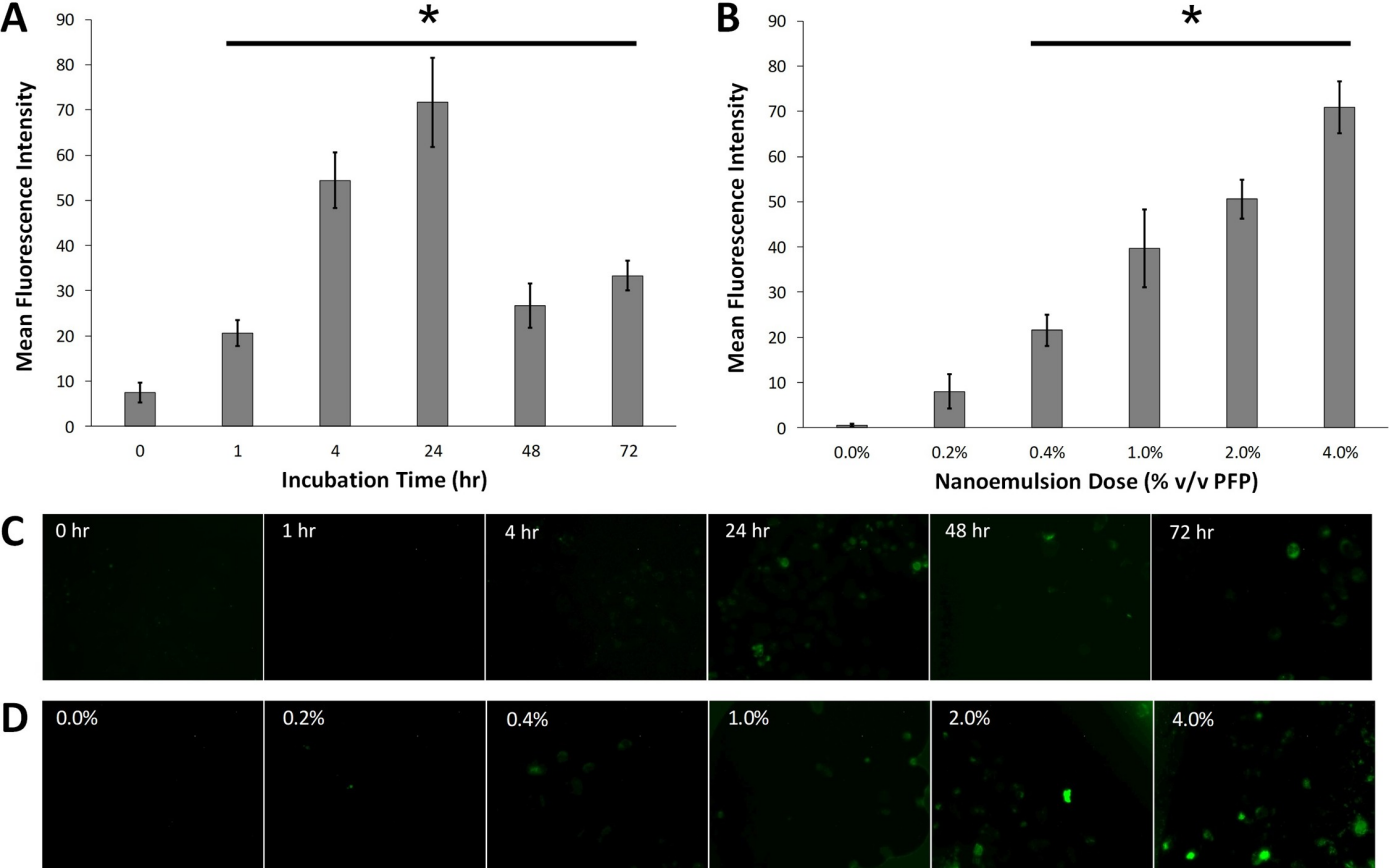
The mean fluorescence intensity of MDA-MB-231 cancer cells incubated with fluorescent AS1411-conjugated nanodroplets as a function of (A) time and (B) dose (%v/v PFP) after 72 hr (n = 10). Representative fluorescence microscopy images at each (C) time point and (D) dose indicate uptake of nanodroplets compared to the initial control sample.

**Fig 5:**
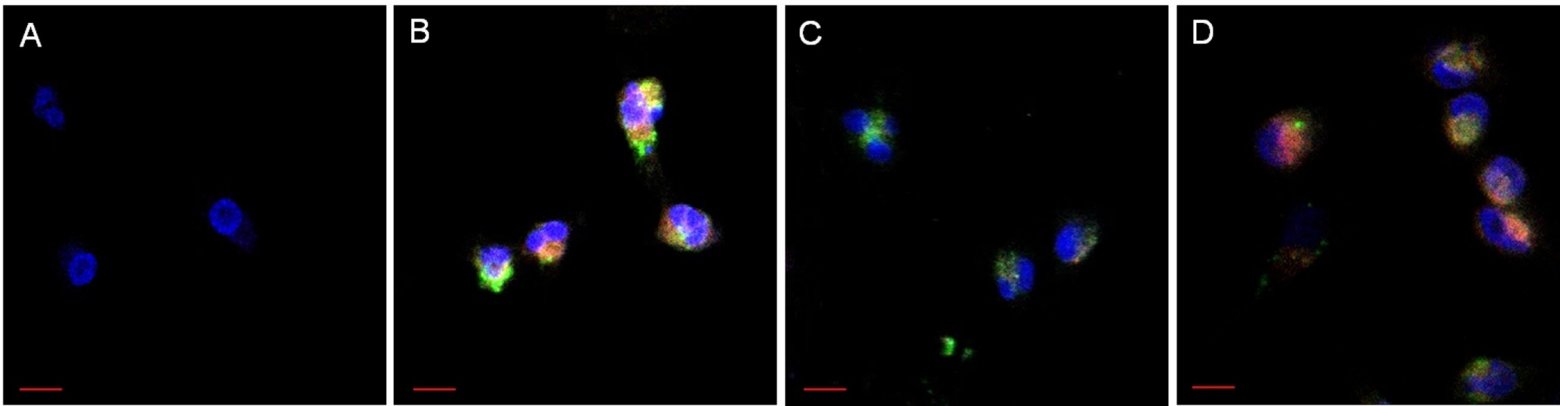
Confocal microscopy images of human breast cancer cells (MDA-MB-231) indicating uptake of fluorescent AS1411-conjugated nanoemulsions. (A) No treatment, (B) 4 hr incubation with AS1411 nanoemulsions, (C) 24 hr incubation with untargeted nanoemulsions, and (D) AS1411 nanoemulsions. (Red: Cy5-labeled AS1411, Green: FITC-labeled lipid, Blue: DAPI nuclear stain). Scale bars represent 10 μm.

Fluorescence microscopy imaging was performed to assess uptake of fluorescent AS1411-conjugated nanodroplets by human breast cancer cells. At a dose of 0.4% PFP (v/v), uptake was detected within 1 hr and persisted for at least 72 hr, with the peak fluorescence intensity detected at 24 hr (Fig 4A). Dose-dependent uptake was also observed at 72 hr (Fig 4B). Furthermore, confocal microscopy imaging indicated uptake and co-localization of fluorescent AS1411-conjugated nanoemulsions in human breast cancer cells (Fig 5). The fluorescence intensity of AS1411 and nanoemulsions in the cells was quantified, indicating rapid uptake by cancer cells within 4 hours.

### Flow cytometry analysis

**Fig 6:**
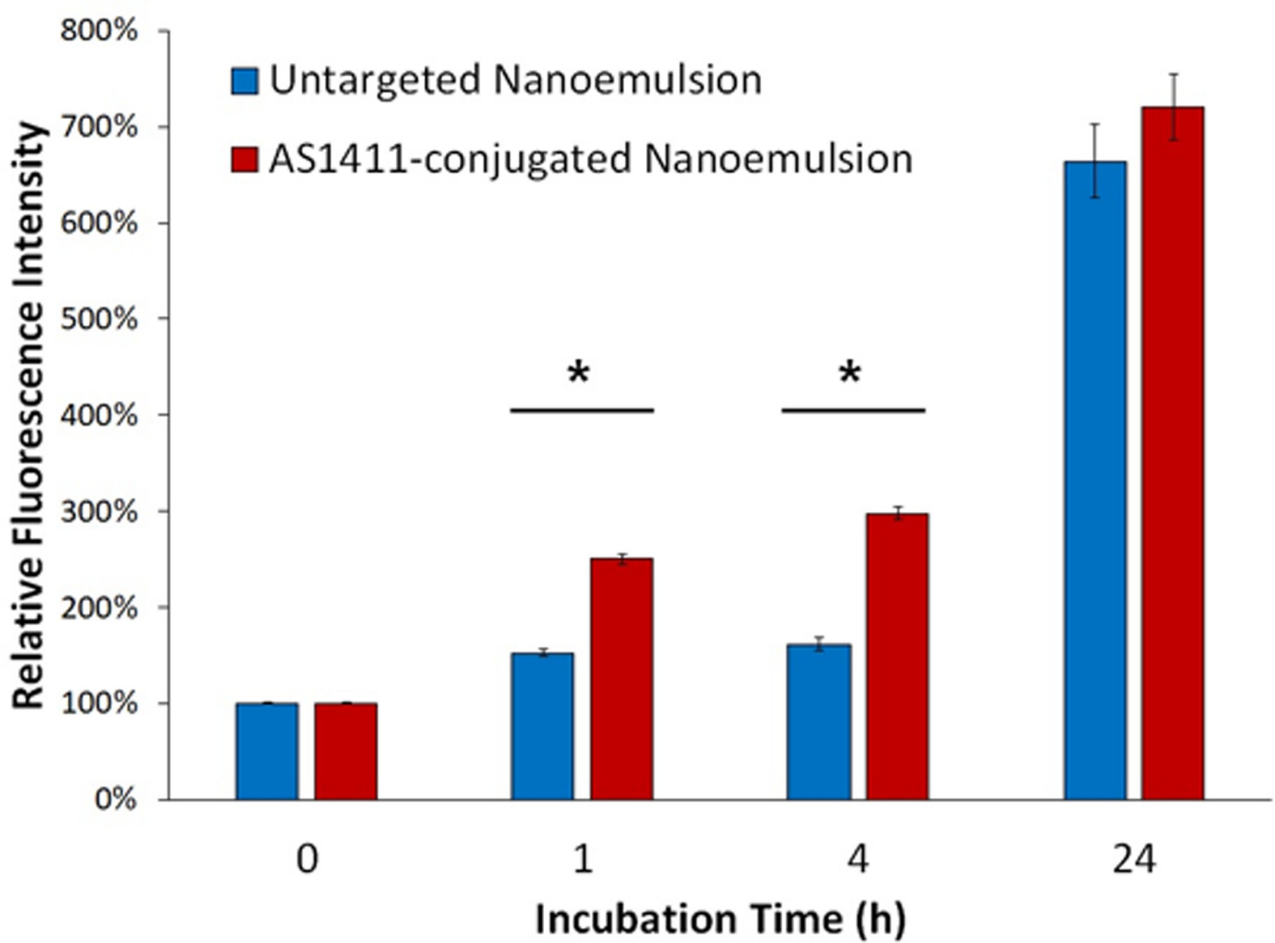
Relative fluorescence intensity of MDA-MB-231 cells after incubation with FITC-labeled nanoemulsions for various time points. Higher uptake was observed in AS1411-conjugated nanoemulsions compared to untargeted emulsions at 1 h and 4 h but there was no significant difference at 24 h (n = 4/group).

Uptake of AS1411-conjugated nanoemulsion compared to untargeted nanoemulsion by MDA-MB-231 cells was assessed using flow cytometry at various time points. As shown in Fig 6, there was a statistically significant increase in uptake of AS1411-conjugated nanoemulsion at 1 h and 4 h compared to untargeted emulsion (p<0.05). The amount of uptake was enhanced at 24 h but there was no significant difference detected between targeted and untargeted nanoemulsion, suggesting that non-specific uptake occurred after longer incubation periods. These results demonstrate that AS1411-conjugated nanoemulsion can enhance uptake by cancer cells, although this effect disappears after long incubation periods.

### Nanoemulsion cytotoxicity studies

**Fig 7:**
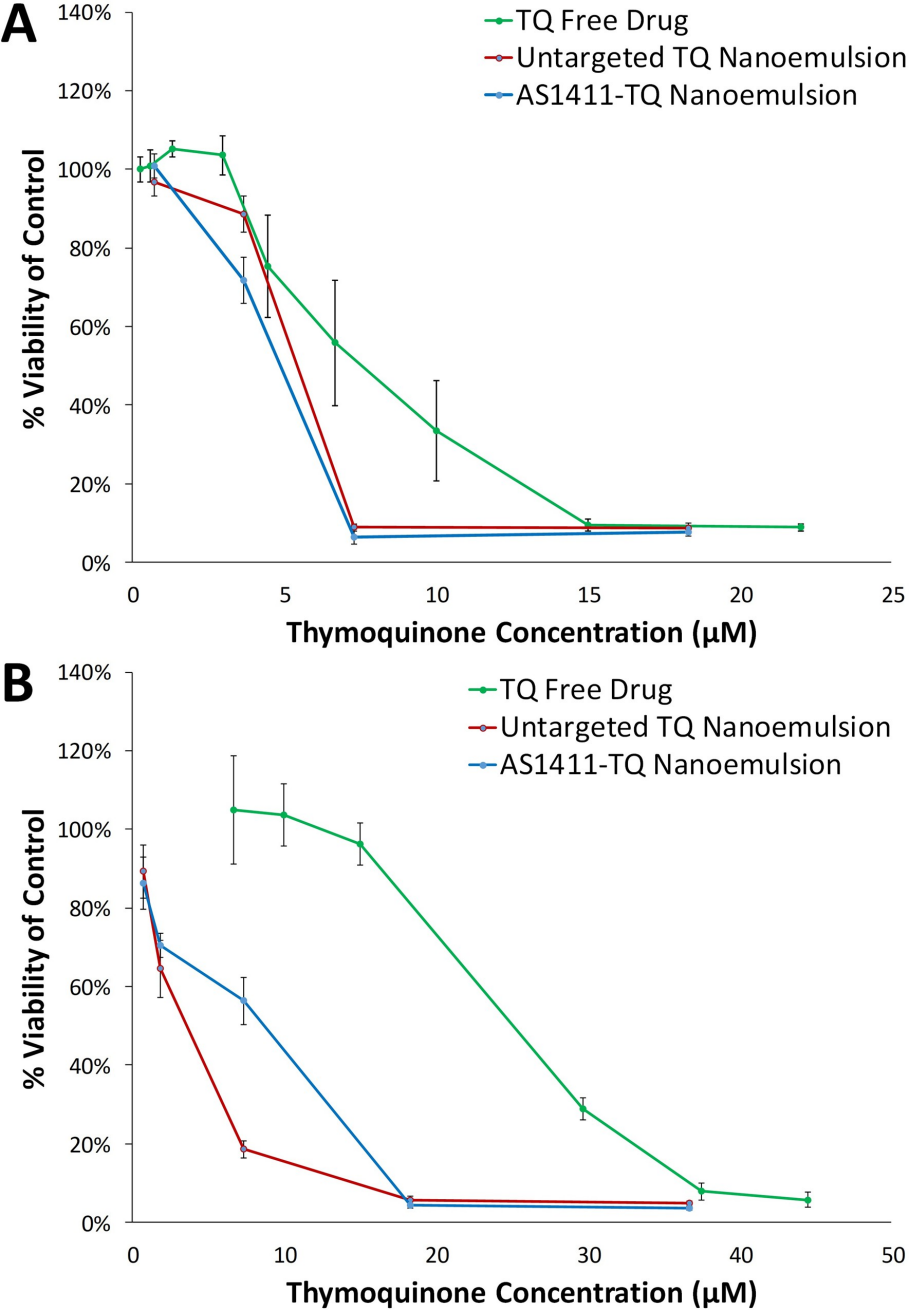
Cytotoxicity of thymoquinone (TQ)-loaded nanoemulsions in two human breast cancer cell lines: (A) MDA-MB-231 and (B) HCC1395, indicating enhanced cytotoxicity of TQ-loaded nanoemulsions compared to free drug (n = 6-13/group). Cytotoxicity was measured with MTT assays.

Cytotoxic effects of AS1411-conjugated nanodroplet emulsions at various concentrations was evaluated in human breast cancer cells (MDA-MB-231 and HCC1395) using MTT assays after 48 hr or 72 hr incubation (Fig 7). Although there was a small (but not statistically significant) increase in cytotoxicity with AS1411 targeting compared to untargeted nanoemulsions in MDA-MB-231 cells, no increase in cytotoxicity was observed with AS1411 targeting in HCC1395 cells. However, both targeted and untargeted nanoemulsions significantly increased cytotoxicity of thymoquinone compared to free compound alone in both cell lines (p < 0.05, n = 6–13).

## Discussion

The results indicate that AS1411 can be conjugated onto thymoquinone-loaded nanodroplet emulsions for targeted delivery to human cancer cells. Aptamers have been explored as targeting moieties to enhance chemotherapy delivery [25], and AS1411 has been tested in clinical trials due to its tumor-targeting and anti-cancer properties. Previous studies utilized AS1411 to target other particles to cancer cells, including gold nanoparticles [19–21], liposomes [26, 27], micelles [28, 29], and other nanoparticle formulations [30, 31]. Drug delivery vehicles have been developed to induce spontaneous release proximal to cancer cells, typically through pH or temperature sensitivity, but these drug delivery vehicles lack in the ability to alter the cell membrane to further enhance uptake. A potential advantage of this nanoemulsion formulation is the ability to enhance intracellular delivery of molecular compounds by transient perforation via acoustic or optical vaporization of perfluorocarbon nanodroplets in tumors [11–15].

Small changes in the average nanodroplet size were measured over time using dynamic light scattering (Fig 3). The concentration of smaller nanodroplets (< 100 nm) decreased at 24 h and 48 h, which may be explained by coalescence or Ostwald ripening effects. The concentration of larger droplets (> 400 nm) increased at 24 h which is also consistent with coalescence or Ostwald ripening [32]. However, the concentration of larger droplets decreased slightly at 48 h, which would not expected to occur by coalescence or Ostwald ripening. Instead, this effect may be due to the increased settling rate for larger droplets, especially given the increased density of the perfluorocarbon core [33]. However, despite the small changes in size, these results indicate that the nanoemulsion was stable in storage during the 48 h observation period. In fact, the size distribution was more monodisperse at 48 h compared to 0 h. The expected shelf-life of this product could be as long as 18 months, based on the shelf-life of a similar room temperature-stable perfluorocarbon emulsion-based product (Oxygent) previously used as an oxygen carrier [34].

Thymoquinone, a natural hydrophobic phytochemical compound with bioactivity in cancer cells, was loaded in nanodroplet emulsions in this study. However, other hydrophobic drugs could also be loaded with this method, including paclitaxel and cyclophosphamide. In addition, hydrophilic drugs, such as cisplatin or doxorubicin, could be loaded into a similar formulation consisting of double emulsions with an aqueous inner phase [35, 36] for targeted tumor delivery. For *in vivo* applications, the dose of therapeutic agents could be controlled by adjusting the nanoemulsion concentration administered or by reducing the amount of drug loaded on each nanodroplet.

In this study a significant difference in uptake was observed between AS1411-conjugated nanoemulsion and untargeted nanoemulsion after incubation between 1–4 h. However, incubation for 24 h or longer resulted in a significant amount of uptake by cancer cells even with untargeted nanoemulsions. This is likely a limitation of the *in vitro* experimental setup, in which the nanoemulsions are in contact with the cells for extended duration of time and may be taken up through endocytosis independent of specific receptors. It is anticipated that this non-specific uptake would be reduced *in vivo* where there is more perfusion around the cells and nanodroplets may be in contact with cells for shorter amounts of time. Future *in vivo* studies will assess the efficacy of this approach in preclinical cancer models. Significant uptake and cytotoxicity of thymoquinone-loaded nanoemulsions was observed in multiple human cancer cell lines, suggesting that AS1411-conjugated nanodroplet emulsions could be an effective formulation for targeted molecular delivery to a variety of different tumor types. Although further development is needed for translation of this approach to clinical use, these results indicate that this formulation could enhance chemotherapeutic delivery to cancer cells and reduce off-target effects by utilizing a target moiety and vaporization approach.

## Conclusions

This study demonstrates that AS1411 can be conjugated onto thymoquinone-loaded nanodroplet emulsions for targeted delivery of molecular compounds to human cancer cells. Furthermore, the AS1411-conjugated nanoemulsion can enhance uptake and cytotoxicity in cancer cells compared to delivery of compounds without nanoemulsion. This formulation may offer significant potential for targeted delivery of chemotherapeutics to tumors for cancer treatment.

## Supporting information

S1 Data (XLSX)
